# Clinical exome sequencing identifies novel gene variants associated with ischemic stroke in the Saudi Tabuk population

**DOI:** 10.3389/fnhum.2025.1645598

**Published:** 2026-01-27

**Authors:** Abdullah Hamadi, Rashid Mir, Osama M. Al-Amer, Mohammed Alasseiri, Waseem AlZamzami, Sael Alatawi, Mohammad A. Alanazi, Mamdoh S. Moawadh, Atif Abdulwahab A. Oyouni, Abeer Al Tuwaijri, Saleh Althenayyan, Hassan A. Madkhali, Raed Alserihi

**Affiliations:** 1 Faculty of Applied Medical Sciences, Department of Medical Laboratory Technology, University of Tabuk, Tabuk, Saudi Arabia; 2 Prince Fahad Bin Sultan Chair for Biomedical Research, University of Tabuk, Tabuk, Saudi Arabia; 3 Department of Biology, FAS, Genome and Biotechnology Unit, Faculty of Science, University of Tabuk, Tabuk, Saudi Arabia; 4 KAIMRC, King Saud Bin Abdulaziz University for Health Sciences (KSAU-HS), Ministry of National Guard Health Affairs (MNG-HA), Riyadh, Saudi Arabia; 5 Clinical Laboratory Sciences Department, College of Applied Medical Sciences, King Saud Bin Abdulaziz University for Health Sciences (KSAU-HS), Riyadh, Saudi Arabia; 6 Department of Pharmacology and Toxicology, College of Pharmacy, Prince Sattam Bin Abdulaziz University, Al-Kharj, Saudi Arabia; 7 Department of Medical Laboratory Sciences, Faculty of Applied Medical Sciences, King Abdulaziz University, Jeddah, Saudi Arabia; 8 Hematology Research Unit (HRU), King Fahad Medical Research Center (KFMRC), King Abdulaziz University, Jeddah, Saudi Arabia

**Keywords:** ischemic stroke, targeted whole-exome sequencing, gene polymorphisms, personalised medicine, MTHFR, KLF14, eNOS, ACE

## Abstract

**Background:**

Variants linked to the risk of ischemic stroke have been discovered through genome-wide association studies (GWASs). These variations frequently have little consequences that lack apparent biological significance. Hence, these findings demonstrate that exome sequencing can be highly relevant to stroke, even though stroke is a complex phenotype with various diseases and risk factors.

**Methodology:**

In this case-control investigation, we used ARMS genotyping to investigate the distribution of polymorphic variations in genes associated with stroke susceptibility. In addition to examine the novel gene variations associated with ischemic stroke we utilized the Illumina NovaSeq 6000 platform for whole-exome sequencing (WES).

**Results:**

Results identified 11 novel gene variants in the GSTT4 gene by targeted whole-exome sequencing, including one deletion GSTT4p.Asn232LysfsTer6, one insertion c.688_689insCG, and 9 SNVs c.699 T > C, c.701C > G, c.708G > T, c.710 T > G, c.712A > G, c.712A > G, c.718A > T, c.719G > A, c.721A > T, c.722G > T in the ischemic stroke patients. We also identified several rare, intermediate, and most common gene variants in cholesterol associated genes LDLR, LDLRAD2, LDLRAD3, APOA2, APOA3, APOA4, APOA5, and PCSK9. Also, several common gene variants were reported in MTHFR, KLF14, eNOS3, and ACE by whole-exome sequencing. Furthermore, the eNOS3-GG and eNOS3-GT genotypes were associated with susceptibility to ischemic stroke (OR = 1.95, *p* < 0.05).

**Conclusion:**

This case-control study identified 11 novel GSTT4 variants and several known polymorphisms associated with ischemic stroke risk in Saudi patients. These findings highlight population-specific genetic factors that warrant further functional and large-scale validation.

## 1 Introduction

A stroke is a brain disorder with a significant cause of death worldwide (Kuriakose and Xiao, 2020). Some relatively heartbreaking and disheartening statistics regarding how the condition impacts individuals worldwide were revealed by the Global Burden of Disease (GBD) research on stroke (Collaborators GBDS, 2021). The Kingdom of Saudi Arabia is a country that is changing quickly and has changed a lot in the last 20 years. Significant changes in living and the environment have increased the risk and number of strokes. In Saudi Arabia, 43, 8 out of every 100,000 people have a stroke (Bakraa et al., 2021). In general there are two types of strokes, the hemorrhagic and ischemic strokes (Boehme et al., 2017). More than 80% of the cases are classified as ischemic stroke, however, the incidence of hemorrhagic vs. the ischemic stroke differs among various populations (Boehme et al., 2017). Hypercholesterolemia can cause plaque to build up on the walls of the arteries, making them narrower increased shear forces in the blood flow leading to platelets deposition and blood clot development (Menet et al., 2018). It is called atherosclerosis, and it can stop blood from flowing through the arteries or cause blood clots, leading to an ischemic stroke (Menet et al., 2018). Low-density lipoprotein receptor (LDLR), apolipoprotein B (APOB), and proprotein convertase subtilisin/kexin 9 (PCSK9) are the three genes most frequently associated with elevated blood cholesterol. In Western and Eastern nations, LDLR accounts for 80–85% of all cases of mutant protein (Futema et al., 2014). Currently, the ClinVar library has over 3,000 different types (Alves et al., 2014). Few people still have different genotypes, such as LDLRAP1 (Ilinca et al., 2020). Numerous studies revealed a connection between elevated LDL-C blood levels and increased coronary artery disease and ischemic stroke risk (Nazir et al., 2020; Hackam and Hegele, 2019). However, the all genes that result in elevated LDL-C and increase the person’s risk for ischemic stroke are not fully demonstrated.

Variants linked to the risk of ischemic stroke, diabetes mellitus, cancer, and cardiovascular disease have been found by genome-wide association studies (GWASs) (Alzahrani et al., 2022; Jalal et al., 2023; Mir et al., 2021, 2022a). These variations frequently have minor consequences that lack evident biological importance. These findings demonstrate that exome sequencing can be very relevant to stroke, even though stroke is a complex phenotype with various diseases and risk factors (Chang et al., 2023). Multiple members of two pedigrees with embolic stroke of unknown origin were subjected to whole-exome sequencing, which revealed probable pathogenic gene variants not previously related to stroke GPR142: c.148C > G (p.Leu50Val) and PTPRN2: c.2416A > G (p.Ile806Val); LRRC1 c.808A > G (p.Ile270Val), SLC7A10c.1294dupG (p.Val432fs), IKBKB: c.1070C > T (p.Ala357Val), and OXGR1 c.392G > A (p.Arg131His), respectively (Hartl et al., 2023). Exome sequencing reveals predicted protein-altering gene variants that may significantly impact ischemic stroke vulnerability. Pathogenic variations in the stroke-related genes NSD1, PKHD1, HRAS, and ATP13A2 have been found (Kumar et al., 2022). These findings confirm that exome sequencing can be crucial in stroke, although it is a complex phenotype with various pathologies and risk factors.

Previous studies have shown that a high amount of homocysteine is linked to aging and neurodegenerative CNS disorders (Dionisio et al., 2010). The metabolic association between homocysteine and the MTHFR gene variation is homocysteine. The MTHFR C677T genotype has been associated with hyperhomocysteinemia in various populations (Kelemen et al., 2004). The MTHFR gene variation has been associated with an increased risk of ischemic stroke in previous studies (Zhao et al., 2022; Kim et al., 2017; Rutten-Jacobs et al., 2016), Moreover, and a strong association between the MTHFR 677C > T SNP and an increased risk of coronary artery disease (CAD) has also been reported in Saudi population (Mir et al., 2022a).

Krüpple-like factors (KLFs) is a family of zink finger transcription factors involved in several biological functions for example regulation of mammalian neuronal differentiation, metabolism, transcription (Avila-Mendoza et al., 2020; Akash et al., 2023; Mir et al., 2022b). They are crucial for maintaining neurons’ health and functions (Yin et al., 2015). According to several KLFs, they control vascular endothelial dysfunction and brain damage in ischemic stroke as transcriptional mediators (Yin et al., 2015; Park et al., 2014). KLF14 rs972283 G > A SNP was reported to be associated with CAD and type 2 diabetes (Gao et al., 2016; Mir et al., 2022b; Elfaki et al., 2023).

The RAS (renin-angiotensin system) functions critically with the angiotensin-converting enzyme (ACE). The RAS (renin-angiotensin system), which helps maintain the fluid-volume balance and blood pressure, includes the angiotensin-converting enzyme (ACE) (Kanugula et al., 2023). It switches bradykinin off and converting angiotensin I into angiotensin II, a vasoconstrictor (Ghebrehiwet et al., 2024). RAS may become a risk factor for heart disease and stroke due to polymorphisms in the gene that codes for ACE (Kato et al., 2011). The ACE insertion/deletion polymorphism may alter the likelihood of an ischemic stroke (Zhang et al., 2012). A crucial component in regulating blood flow, inflammation, neurogenesis, and blood coagulation is endothelial nitric oxide synthase (eNOS) (Shyu et al., 2017). After an ischemic stroke, it aids in reducing immediate ischaemia damage and protecting nerve cells. Additionally, because eNOS knockout animals have been demonstrated to have impaired sensory-motor function and poor spatial memory, it has evolved into a crucial method for the brain to recover from ischemia injury. An increased risk of stroke and CAD is associated with specific genotypic polymorphisms of eNOS (Mir et al., 2022a; Heidar and Khatami, 2017).

Whole-exome sequencing, combined with targeted stroke gene panels, can be used to identify rare monogenic forms of stroke. It is essential to carefully assess the clinical significance and potential pathogenicity of newly detected variants. In our study, most stroke patients with a family history of stroke did not have a previously known genetic cause. Screening using whole-exome sequencing and a comprehensive stroke gene panel may therefore reveal rare and novel variants associated with stroke susceptibility.

Despite the increasing incidence of ischemic stroke in Saudi Arabia, few studies have examined population-specific genetic risk factors. Addressing this gap, the present study investigates key variants in the Tabuk population, providing insights essential for developing personalized prevention and treatment strategies. To our knowledge, no previous study has explored the frequency or potential association of novel, rare, or intermediate gene variants with stroke in the Tabuk population.

## 2 Methodology

### 2.1 Study population

This study followed an unmatched case-control design to investigate genetic and clinical risk factors associated with ischemic stroke among Saudi men and women in middle age. A total of 220 participants were recruited, comprising 105 ischemic stroke patients (cases) and 115 healthy controls from the Tabuk region of Saudi Arabia. The study was conducted in accordance with the STROBE (Strengthening the Reporting of Observational Studies in Epidemiology) guidelines to ensure methodological transparency and reproducibility. All stroke cases were clinically diagnosed based on neuroimaging and clinical criteria, while controls had no history of cerebrovascular disease. Only Saudi nationals were included in the study; non-Saudi participants were excluded.

#### 2.1.1 Recruitment sites

All stroke patients were recruited in the neurology departments of King Khaled Hospital and King Salman Military Hospital in Tabuk, Saudi Arabia. The healthy control group was made up of patients who went to King Salman Military Hospital and King Khaled Hospital in Tabuk for regular examinations. These subjects completed both the questionnaire and the informed consent form.

#### 2.1.2 Ethics approvals

The University of Tabuk’s Ethics Committee approved the research study’s ethical approval (UT-91-23-2020). The study was conducted per the established guidelines for employing human subjects in research, including the Helsinki Declaration principles. Informed consent was obtained before collecting samples from either patients or controls.

#### 2.1.3 Data collection from stroke patients

A standardized questionnaire for hospitalized patients was used to collect information on demographics, such as age and sex, and cerebrovascular risk factors, such as systemic hypertension (two outpatient blood pressure values over 140/90 mmHg). A standardized questionnaire was used to collect information on patient admission, including demographic information like age and sex and cerebrovascular risk factors such as systemic hypertension. Other factors include diabetes mellitus, atrial fibrillation found during any previous monitoring, dyslipidemia (defined as total serum cholesterol above 200 mg/dL, LDL above 100 mg/dL, HDL above 50 mg/dL, or triglycerides above 150 mg/dL), and current smoking status.

#### 2.1.4 Sample collection from stroke patients

Each stroke patient had approximately 3 mL of peripheral blood drawn and placed in an EDTA or Lavender top tube. All healthy control samples were drawn simultaneously as part of a routine blood draw for exercise. It is indicated that no more blood tests were required. The blood specimens were kept between −20 °C and −30 °C.

### 2.2 Whole exome sequencing protocol

#### 2.2.1 Genomic DNA extraction

Per the manufacturer’s instructions, DNA was extracted from peripheral blood samples from clinically verified stroke cases and healthy controls using the DNeasy Blood Kit (Qiagen, Hilden, Germany). After that, the DNA was combined with water devoid of nuclease and stored at 4 °C.

#### 2.2.2 Quality and integrity of DNA

Using the optical density (OD) at A260 and A280 was evaluated using NanoDropTM (Thermo Scientific, Waltham, MA, USA) to assess the caliber and integrity of the DNA extracted from the stroke samples. The A260/A280 ratio was in the range of 1.80 and 1.96, indicating acceptable DNA quality. On 1% gels, electrophoresis revealed that the genomic DNA was sound and complete. On highly high-quality DNA samples, whole exome sequencing was carried out.

#### 2.2.3 Library preparation

Exome sequencing, also called whole exome sequencing (WES), is a way to sequence the parts of a genome that code for proteins. The twist is that Exome 2.0 has the best performance in its class and covers the most protein-coding and noncoding parts of the genome.

#### 2.2.4 The twist 2.0 exome kit’s

Exome kit is made up of During both the library preparation and sequencing steps, the user instructions for the Illumina NovaSeq 6000 platform was used. FastQC v0.11.9 was used to check the quality of the sequencing reads.

#### 2.2.5 TrimGalore v0.6.6

Low-quality bases and sequencing adapters were taken out of raw results with TrimGalore v0.6.6. High quality (HQ) readings were used to map the reads onto the hg38 human reference genome. The GATK (v4.2.4.1) best practice system and the haplotype caller were used to call variations [single-nucleotide variants (SNVs), small InDels]. Different libraries and tools were used to add notes to the variants.

#### 2.2.6 RefSeq database

Gene-related variation was discovered and discussed using the RefSeq database. We investigated whether the differences were associated with the disorders using databases such as OMIM and ClinVar. ExAC, GnomAD exome, GnomAD genome, and ESP were used to remove variation and frequent variations. Also utilized were population frequency data from 1,000 genomes. The impact of the coding non-synonymous SNVs on the protein’s structure and function was assessed using PolyPhen-2 and the SIFT score. Many prediction algorithms also looked at each variant separately for the in-silicon variation effect prediction. Each variant was classified into one of three groups based on ACMG recommendations (25741868): pathogenic, potentially pathogenic, or variants of unknown importance.

### 2.3 Genotyping of KLF14, eNOS3- Glu298Asp., MTHFR, ACE- I/D

*Amplification-refractory mutation system PCR*: ARMS-PCR was used for Krüppel-like factor 14 rs972283 G > A, eNOS3- rs1799983 (Glu298Asp) G > T, MTHFR 677 C > T genotyping, and mutation-specific PCR was used for ACE- rs4646994 I/D genotyping (Table 1). The primers used in the studies have been described before, and our lab optimized the experimental methods to match (Mir et al., 2021, 2022a).

**Table 1:** Primers for KLF14 rs972283 G > A, MTHFR 677 C > T, ACE- rs4646994 I/D, and eNOS3- rs1799983 G > T gene variation.

Direction	Sequence	Product size	AT
**ARMS primers of KLF14 rs972283 G > A genotyping**			
KLF14 Fo	5′-GTCATAGGTCAAACAGCTAGATATTGGGT-3′	437 bp	60°C
KLF14Ro	5′-TCTACAGGACCAACTCAAATTATGAGGT-3′		
KLF14 FI (G allele)	5′-TCATTGTATACTTGGAAAAAATCCTACATG-3′	274 bp	
KLF14 RI (A allele)	5′-TATGTAAAAATAAGTATGCGCCATGCCT-3′	221 bp	
**Mutation specific *PCR* primers of ACE- rs4646994 I/D genotyping**			
ACE-F	5′-CTGGAGACCACTCCCATCCTTTCT-3′	490-bp (II)	58 °C
ACE-R	5′-GATGTGGCCATCACATTCGTCAGAT-3′.	190-bp (DD)	
**ARMS primers of MTHFR 677 rs1801133 C > T genotyping**			
MTHFR-677Fo	5′-AAGCATATCAGTCATGAGCCCAGCC-3′,	224 bp	58°C
MTHFR-677Ro	5′-GGGAAGAACTCAGCGAACTCAGCAC-3′		
MTHFR-677FI-C	5′-AGGAGAAGGTGTCTGCGGGCGT-3′	101 bp	
MTHFR-677RI-T	5′-AAGAAAAGCTGCGTGATGATGAAATAGG-3′	177 bp	
**ARMS primers of eNOS3- rs1799983 G > T genotyping**			
eNOS-FO	5′-AGCCTCGGTGAGATAAAGGATG-3′	701 bp	60°C
eNOS-RO	5′-CCTGGACCTGCTCTGATTGTC-3′		
eNOS-FI-G	5′-GCTGCTGCAGGCCCCAGATAAG-3′	475 bp	
eNOS-RI-T	5′-GCAGAAGGAAGAGTTCTGGGAGA-3′	271 bp	
Primers for sanger sequencing			
**Sanger sequencing primers KLF14 rs972283 G > A**			
KLF14-F	5′-CTCCTCCCCATTTCTCATCA-3′	297 bp	59 °C
KLF14-R	5′-CCAAGAAAATACAAAGAGGAAAGG-3′		
**Sanger sequencing primers MTHFR677rs1801133C > T**			
MTHFR677	5′-CAAGCAACGCTGTGCAAGTTCTGG-3′	480 bp	58°C
MTHFR677	5′-TGTGCTGTGCTGTTGGAAGGTGCA-3′		
**Sanger sequencing primers eNOS 894G > T rs1799983**			
Glu298Asp	5′-AAGGCAGGAGACAGTGGATGGA-3′	248-bp	62 °C
Glu298Asp	5′-CCCAGT CAATCCCTTTGGTGCTCA-3′		

*PCR reactions and assay conditions*: Template DNA (50 ng), FO -0.12 ul, RO -0.12 ul, FI -0.12 ul, RI -0.12 ul (25 pmol of each primer), and 6 ul of Green PCR Master Mix (2X) (Cat# M712C, Promega, USA) were used in the PCR experiments. The final volume was adjusted to 12 ul with the addition of nuclease-free ddH2O.

*Thermocycling conditions*: The thermocycling conditions used were at 95 °C for 6 min followed by 33 cycles of 95 °C for 32 s, 60 °C for KLF14 rs972283 G > A, 60.5 °C for eNOS3- rs1799983 (Glu298Asp) G > T, 58 °C for MTHFR 677 C > T rs1801133 and 58 °C for ACE- rs4646994 I/D, for 40 s which was followed by the final extension at 72 °C for 12 min and storage at 72 °C for 12 min.

*Gel electrophoresis and PCR product visualization*: 2% agarose gel electrophoresis was used to separate the PCR products, and they were then dyed with Sybre safe dye (Thermo Scientific, Waltham, MA, USA). The Gel documentation system from Bio-Rad (Hercules, California, USA) was used to visualize the gel picture.

*KLF14 rs972283 G > A*: Outer primers Fo and Ro expand the outer part of the KLF14, which makes a 437-bp band used to check the purity of the DNA. Primers Fo and RI make a band of 221 bp from the A allele, and Fo and RI make a band of 274 bp from the G allele.

*ACE- rs4646994 I/D*: I/I = (490-bp fragment), D/D = (190-bp fragment), and I/D = (both 490- and 190-bp fragments) were used for the ACE- rs4646994 I/D amplification.

*MTHFR 677 rs1801133 C > T*: Primer exteriors MTHFR 677 C > T’s outer region is amplified by FO and RO, creating a band of 224 bp that serves as a DNA purity checker. A band of 177 bp is produced by the T allele using primers FO and RI, whereas a band of 101 bp is produced by the C allele using primers FI and RO.

*eNOS3- rs1799983 (Glu298Asp) G > T*: Primer exteriors the eNOS3rs1799983G > T’s outer region is amplified by FO and RO, creating a band of 701 bp that serves as a DNA purity control. A band of 271 base pairs (bp) is produced by the T allele using primers FO and RI, and a band of 475 bp is produced by the G allele using primers FI and RO.

#### 2.3.1 Sanger sequencing for the confirmation of genotyping results

**Figure 1:**
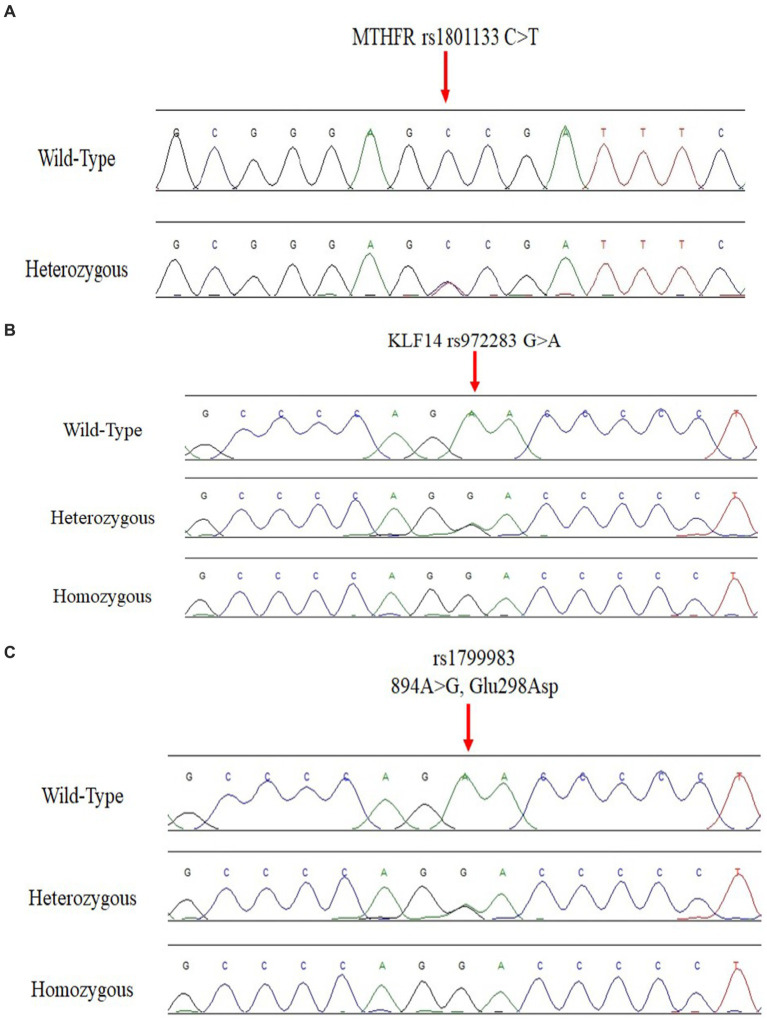
**(A)** *MTHFR* 677 C > T genotyping by Sanger sequencing. **(B)** KLF14 rs972283 G > A genotyping by Sanger sequencing. **(C)** eNOS3- rs1799983 (Glu298Asp) G > T genotyping by Sanger sequencing.

To confirm the genotyping results of Krüppel-like Factor 14 rs972283 G > A, eNOS3- rs1799983 (Glu298Asp) G > T, *MTHFR* 677 C > T genotyping detected by ARMS-PCR, 20 randomly selected PCR products from the PCR systems for polymorphic sites in these gene were sequenced using a sanger sequencing. Two primers F seq and R Seq were used as sequencing primers as depicted in Table 1 for the detection of the genotyping in the above genes. The PCR amplification was done followed by purification using QIAquick PCR Purification Kit from Qiagen (Germany). Finally, the purified PCR products were sequenced by Applied Biosystems sequencer as depicted in Figure 1A (*MTHFR* 677 C > T), Figure 1B, KLF14 (rs972283 G > A), and Figure 1C, eNOS3- rs1799983 (Glu298Asp) G > T.

### 2.4 Statistical analysis

The Deviations from the Hardy–Weinberg equilibrium (HWE) were assessed using the chi-square (χ^2^) goodness-of-fit test. Group differences were analyzed using the student’s *t*-test or one-way analysis of variance (ANOVA) for continuous variables, and the chi-square test for categorical variables. The associations of KLF1-4 rs972283 G > A, MTHFR 677 C > T, ACE- rs4646994 I/D, and eNOS3 rs1799983 (Glu298Asp) G > T genotypes between stroke patients and controls were determined using chi-square tests. A *p*-value ≤ 0.05 was considered statistically significant. All statistical analyses were performed using SPSS version 16.0 (SPSS Inc., Chicago, IL, USA). All methodological and reporting steps adhered to the STROBE (Strengthening the Reporting of Observational Studies in Epidemiology) checklist for observational studies.

## 3 Results

This study identified 11 novel variants in the GSTT4 gene (one deletion, one insertion), nine SNVs, and several known variants in cholesterol- and vascular-associated genes (LDLR, LDLRAD2, LDLRAD3, APOA2, APOA3, APOA4, APOA5, PCSK9, MTHFR, KLF14, eNOS3, and ACE). The alleles A of KLF14, T of MTHFR, T of eNOS3, and D of ACE were potentially associated with increased susceptibility to ischemic stroke in the Tabuk population. These findings collectively suggest that oxidative stress, endothelial dysfunction, and lipid metabolism pathways play essential roles in stroke pathogenesis among Saudi patients. Moreover, our whole-exome sequencing results revealed frequently mutated genes previously implicated in stroke, including LDLR, LDLRAD3, LDLRAD2, APOA5, PCSK9, MTHFR, eNOS3, and KLF14.

*Low-density lipoprotein receptor (LDLR)-LDLR rs5927 (c.2232A > G)* is the most prevalent LDLR gene variant found by whole exome sequencing in all of our stroke patients (Table 2).
*LDLRAD3 gene: LDLRAD3 rs1138807 (c.219A > G)* is the most prevalent variation of the gene for Low-density Lipoprotein Receptor Class A Domain-Containing Protein 3 found in our stroke patients, according to WES.
*LDLRAD2 gene*: The variant rs10917051 (c.401A > C) has been found in the LDLRAD2 gene (Table 2). The full exome sequencing of all of our stroke patients revealed the insertion/deletion APOA2 gene variant rs17244502 (c.152-11_152-6del) in the apolipoprotein A2 (APOA2) gene.
The most frequent variant of the APOA3 gene found by WES in our stroke patients is APOA3- rs5092 (c.87G > A) the APOA4 gene (Table 2). APOA4- rs5104 (c.440G > A) is the most prevalent APOA4 gene variant discovered by whole exome sequencing.
*Apolipoprotein A5 (apoA5) gene* is essential for the metabolism of triglycerides (TG) and TG-rich lipids. APOA5- rs2072560 (c.203A > G) was the gene variation most often discovered by whole exome sequencing in our stroke patients (Table 2).
*The enzyme (serine protease) PCSK9, also known as proprotein convertase subtilisin/kexin type 9,* is mainly synthesized in the liver. LDL-R is degraded due to PCSK9’s interaction with it on the surface of hepatocytes, which increases LDL-C levels in the blood. The most frequent PCSK9 gene variants identified by whole exome sequencing in all of our stroke patients were PCSK9- rs509504 (c.1026A > G), PCSK9- rs540796 (c.1380A > G), PCSK9- rs562556 (c.1420G > A), and PCSK9- rs505151 (c.2009G > A) (Table 3).

**Table 2:** Association of various genotypes with gender and age in stroke patients.

I. Stroke patients with Krüppel-like factor 14 rs972283 G > A genotypes							
	*N*	GG	GA	AA	Df	*X* ^2^	*p*-value
Stroke patients	100	28	37	35			
**Association of KLF14 G > A genotypes with gender**							
Male	62	12	28	22	2	7.03	0.026
Female	38	16	9	13			
**Association of KLF15 G > A genotypes with age**							
Age < 50	20	08	05	07	2	2.26	0.33
Age > 50	80	20	32	28			
II. Stroke patients with MTHFR 677 C > T genotypes							
	*N*	CC	CT	TT	Df	*X* ^2^	*p*-value
Stroke patients	103	42	53	08			
**Association of MTHFR 677 C > T genotypes with gender**							
Male	62	20	35	07	2	6.31	0.049
Female	41	22	18	01			
**Association of MTHFR 677 C > T genotypes with age**							
Age < 50	23	12	06	05	2	12.09	0.0024
Age > 50	80	30	47	03			
III. Stroke patients with eNOS3- rs1799983 (Glu298Asp) G > T genotypes							
	*N*	GG	GA	AA	Df	*X* ^2^	*p*-value
Stroke patients	103	38	57	08			
**Association of eNOS3 G > T genotypes with gender**							
Male	62	25	36	1	2	8.3	0.015
Female	41	13	21	7			
**Association of eNOS3 G > T genotypes with age**							
Age < 50	23	08	10	5	2	8.33	0.016
Age > 50	80	30	47	03			
IV. Stroke patients with ACE- rs4646994 I/D genotypes							
	*N*	II	ID	DD	Df	*X* ^2^	*p*-value
**Association of ACE- rs4646994 I/D with gender**							
Male	62	3	16	43	2	8.76	0.012
Female	41	10	7	24			
**Association of ACE- rs4646994 I/D with age**							
Age < 50	23	3	8	12	2	6.82	0.044
Age > 50	80	10	15	55			

**Table 3:** Hypercholesterolemia associated gene variants by whole exome sequencing in stroke patients.

Gene	Chromosome	Base change	dpSNP_RS number	Exon number
LDLRAD3	Chr11	c.219A > G	rs1138807	(3/6)
LDLRAD2	Chr1	c.401A > C	rs10917051	(2/5)
LDLR	Chr19	c.2232A > G	rs5927	(15/18)
APOA5	Chr11	c.203A > G	rs2072560	(3/3)
APOA4	Chr11	c.440G > A	rs5104	(3/3)
APOA4	Chr11	c.87G > A	rs5092	(2/3)
APOA2	Chr1	c.152-11_152-6del	rs17244502	(NA)
PCSK9	Chr1	c.1026A > G	rs509504	(7/12)
PCSK9	Chr1	c.1380A > G	rs540796	(9/12)
PCSK9	Chr1	c.1420G > A	rs562556	(9/12)
PCSK9	Chr1	c.2009G > A	rs505151	(12/12)

*Methylenetetrahydrofolate reductase gene (MTHFR)*: The MTHFR rs750510348 C > T (c.1428C > T), MTHFR- rs2274976 (c.1904G > A), MTHFR rs2066462 (c.1179C > T), and MTHFR rs2066470 (c.240C > T) are the most prevalent variants found by whole exome sequencing in all of our stroke patients (Table 4).

**Table 4:** Distribution of gene variants of KLF14 rs972283 G > A, MTHFR 677 C > T, eNOS3- rs1799983 G > T in stroke patients and controls.

Association of KLF14 rs972283 G > A gene variation between stroke patients and controls									
Subjects	*N*	GG	GA	AA	Df	*X* ^2^	G	A	*p*-value
Cases	100	28 (28%)	37 (37%)	35 (35%)	2	11	0.47	0.53	0.0004
Controls	120	47 (39.16%)	54 (45%)	19 (15.83%)			0.62	0.38	
Association of MTHFR 677 C > T gene variation in Stroke cases and controls									
Subjects	*N*	CC	CT	TT	Df	*X* ^2^	C	T	*p*-value
Cases	103	42 (40.77%)	53 (51.45%)	08 (7.76%)	2	14.28	0.67	0.33	0.0008
Controls	120	79 (65.83%)	37 (30.83%)	04 (3.33%)			0.81	0.19	
Association of eNOS3- rs1799983 G > T gene variation between stroke patients and controls									
Subjects	*N*	GG	GT	TT	Df	*X* ^2^	G	T	*p*-value
Cases	103	38 (36.89%)	57 (55.33%)	08 (7.76%)	2	6.97	0.65	0.35	0.030
Controls	120	65 (54.16%)	50 (41.66%)	05 (4.16%)			0.75	0.25	
Association of ACE I/D gene variation between Stroke cases and controls									
Subjects	*N*	II	DI	DD	Df	*X* ^2^	I	D	*p*-value
Cases	103	13 (12.62%)	23 (22.33%)	67 (65.04%)	2	17.74	0.24	0.76	0.0001
Controls	120	20 (16.66%)	55 (45.83%)	45 (37.5%)			0.60	0.40	

*Endothelial nitric oxide synthase (eNOS3) genes*: All of our stroke patients had NOS3rs1549758 (c.774 T > C), NOS3rs1799983 (c.894 T > G), and NOS3rs2566514 (c.1998C > G) as the most prevalent eNOS3 gene variations, according to whole exome sequencing results (Table 4).

*Krüppel-like factor 14*: All of our stroke patients had the following KLF14 gene variations most often found by whole exome sequencing: KLF14 rs111400400 (c.172C > T), KLF14 rs184537657 (c.140C > A), and KLF14 rs111731678 (c.117 T > A) (Table 4). Additionally, we discovered that the KLF14 missense mutation rs111400400 (Ser58Pro) was linked to stroke.

*Angiotensin converting enzyme (ACE) gene*: All of our stroke patients had the following ACE gene variations most frequently found by whole exome sequencing: ACE- rs4362 (c.3387 T > C), ACE- rs4343 (c.2328G > A), and ACE- rs4309 (c.1215C > T) (Table 5).

**Table 5:** Gene variants detected by whole exome sequencing in stroke patients.

Gene	Chromosome	Variant type	Base change	dpSNP_RS number	Exon number
MTHFR	chr1	SNP	c.1428C > T	rs750510348	(9/13)
MTHFR	chr1	SNP	c.1904G > A	rs2274976	(13/13)
MTHFR	chr1	SNP	c.1179C > T	rs2066462	(8/13)
MTHFR	chr1	SNP	c.240C > T	rs2066470	(3/13)
NOS3	Chr7	SNP	c.774 T > C	rs1549758	(7/27)
NOS3	Chr7	SNP	c.894 T > G	rs1799983	(8/27)
NOS3	Chr7	SNP	c.1998C > G	rs2566514	(17/27)
KLF14	Chr7	SNP	c.172C > T	rs111400400	(1/1)
KLF14	Chr7	SNP	c.140C > A	rs184537657	(1/1)
KLF14	Chr7	SNP	c.117 T > A	rs111731678	(1/1)
ACE	chr17	SNP	c.3387 T > C	rs4362	(23/25)
ACE	chr17	SNP	c.2328G > A	rs4343	(16/25)
ACE	chr17	SNP	c.1215C > T	rs4309	(8/25)

GSTT4 *glutathione S-transferase theta 4-(GSTT4)*: In this protein, an enzyme is active. This reaction is known to be catalyzed by it. A halide anion, S-substituted glutathione, and H (+) are the results of glutathione plus RX. Targeted Whole-Exome analysis results revealed 11 new gene variations in the GSTT4 gene. One insertion GSTT4c.688_689insCG, one deletion GSTT4p.Asn232LysfsTer6 and nine SNVs were sequenced. GSTT4c.699 T>. The following gene variants have been found in ischemic stroke patients: C, GSTT4-c.701C > G, GSTT4-c.708G > T, GSTT4-c.710 T > G, GSTT4-c.712A > G, GSTT4-c.718A > T, GSTT4-c.719G > A, GSTT4-c.721A > T, and GSTT4-c.722G > T in Table 6.

**Table 6:** *GSTT4* novel gene variants detected by whole exome sequencing in stroke patient*s.*

Hugo symbol	Variant classification	Variant class	HGVSc	HGVSp	HGVSp_Short	dbSNP-Rs
GSTT4	Frameshift variant	Deletion	p.Asn232LysfsTer6	c.696del	p.N232Kfs*6	Novel
GSTT4	Frameshift variant	Insertion	c.688_689insCG	p.Lys230ThrfsTer9	p.K230Tfs*9	Novel
GSTT4	Missense variant	SNV	c.722G > T	p.Arg241Met	p.R241M	Novel
GSTT4	Missense variant	SNV	c.719G > A	p.Ser240Asn	p.S240N	Novel
GSTT4	Missense variant	SNV	c.721A > T	p.Arg241Trp	p.R241W	Novel
GSTT4	Missense variant	SNV	c.718A > T	p.Ser240Cys	p.S240C	Novel
GSTT4	Missense variant	SNV	c.712A > G	p.Lys238Glu	p.K238E	Novel
GSTT4	Missense variant	SNV	c.710 T > G	p.Leu237Arg	p.L237R	Novel
GSTT4	Missense variant	SNV	c.708G > T	p.Leu236Phe	p.L236F	Novel
GSTT4	Missense variant	SNV	c.701C > G	p.Ser234Cys	p.S234C	Novel
GSTT4	Missense variant	SNV	c.699 T > C	p.Ile233=	p.I233=	Novel

**Figure 2:**
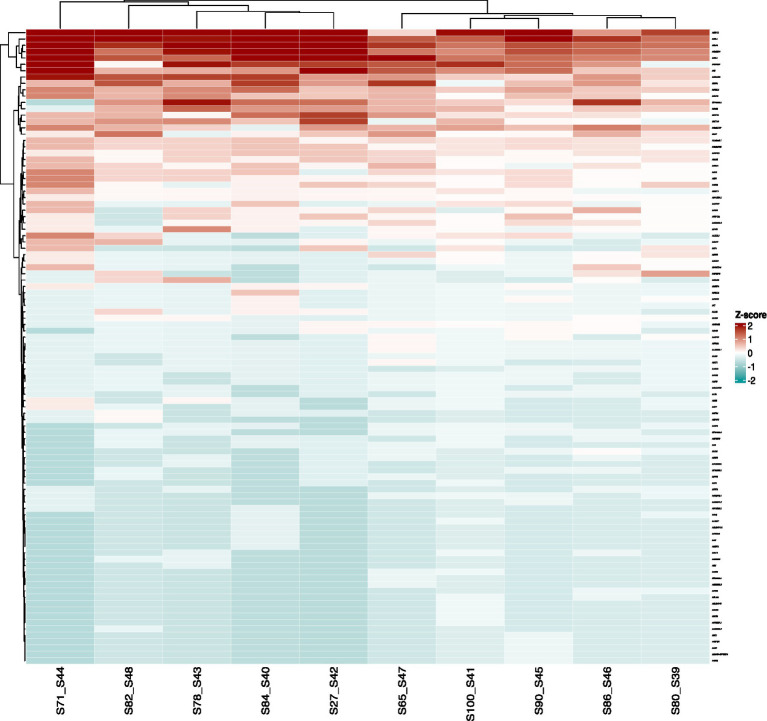
Overview at the distribution of the gene variants (random 100 genes).

To produce a comprehensive overview of the somatic background mutations found in Saudi patients with a history of CES, a compendium of genes known to play a role in coagulation, angiogenesis, and metabolism. Variants per gene were tallied for each of the chosen genes, yielding variants count matrix that was then converted to *Z*-score values and plotted as a heatmap (Figure 2). According to the generated heatmap, we observed a small number of genes with a high number of variants in between more than 50–100% of CES patient samples. Consequently, this observation implies possible common variants that could aid in the diagnosis of CES and pave the way for early therapeutic interventions.

**Figure 3:**
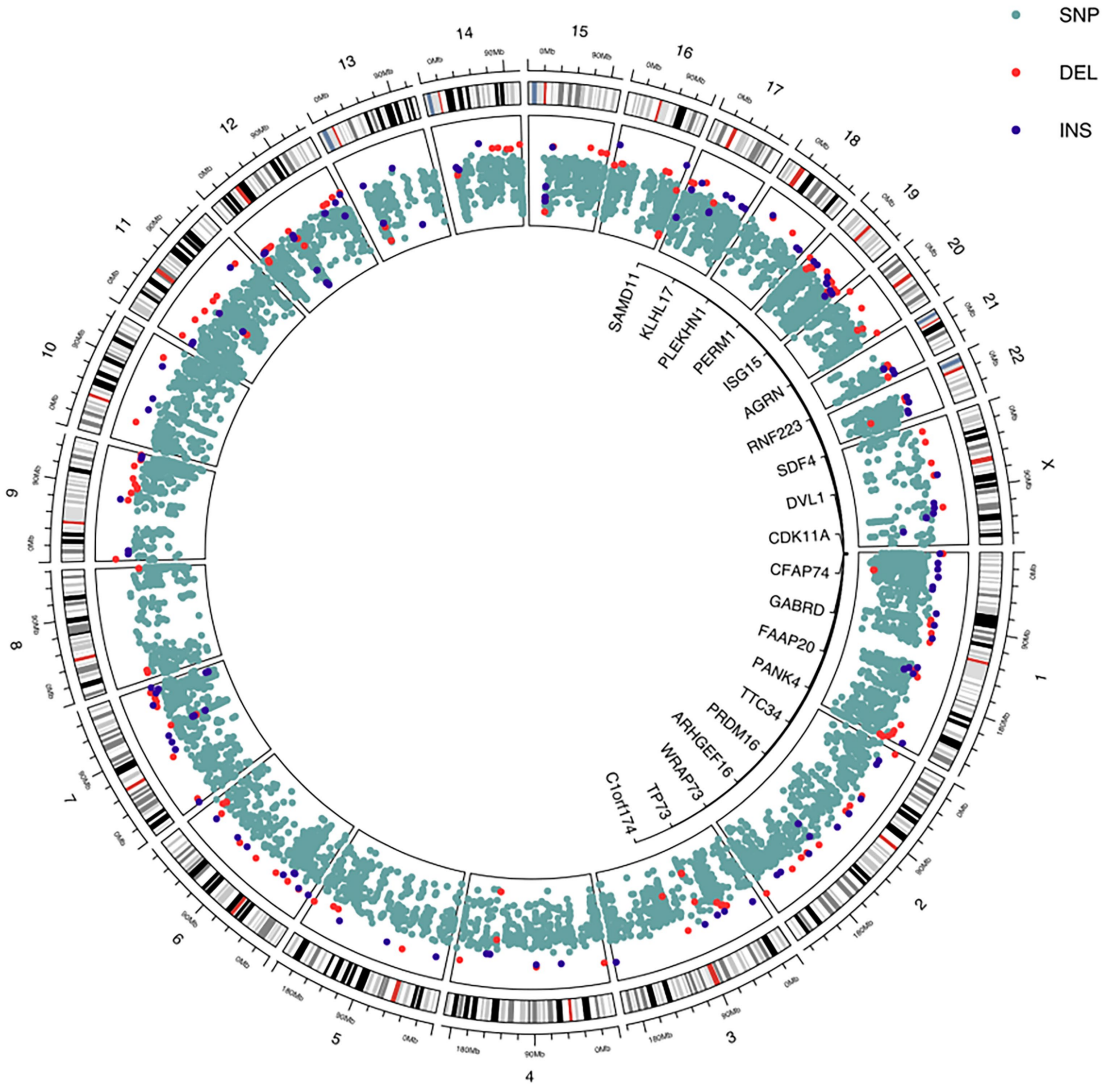
The genome-wide distribution of the identified known gene variants with a frequent distribution of 80% or higher are plotted.

A circular genome plot (Figure 3) for the human genome (hg38) was produced to provide a general overview of the distribution of discovered known variations per chromosome that were present in more than 80% of the samples. Variants were represented by dots, with the colors turquoise denoting SNPs, red denoting DELs, and dark blue denoting INSs. The graphic only includes variants with a frequent distribution of 80% or greater. The figure only included the top 20 genes with known variation numbers. With the exception of chromosome X, we saw increased and evenly distributed SNPs between the chromosomes from the figure. It is important to note that the top 20 genes with known variations are all located on chromosome 1.

**Figure 4:**
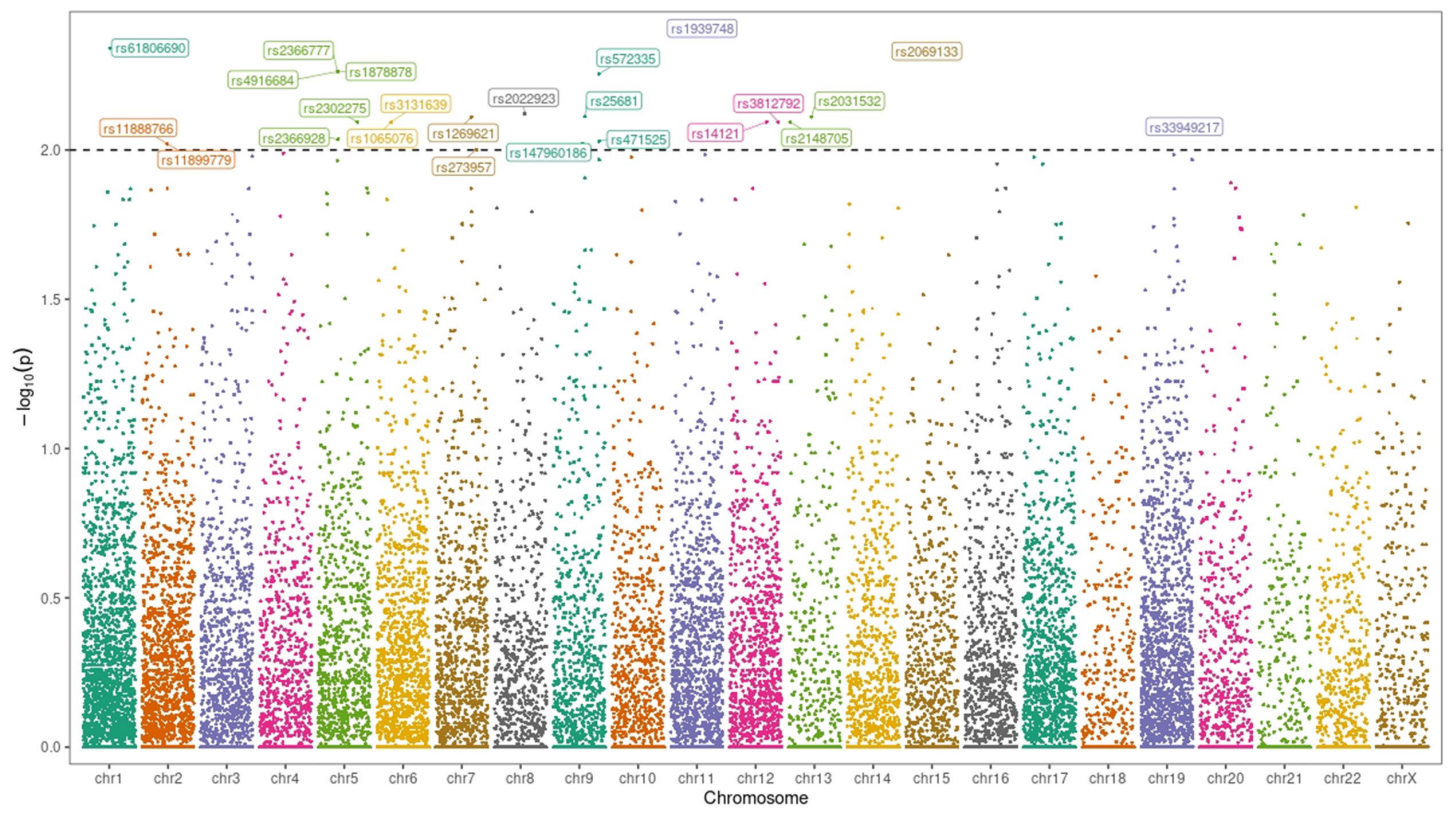
Clinical characteristics of the study population and correlation of patients’ genotype with gender and age.

In Figure 4, genome-wide association studies (GWAS) are a method used to identify genetic variants linked to specific traits or diseases. This analysis involves processing genetic data to extract genotype information from multiple samples. Data from both case and control groups are cleaned and combined into a comprehensive matrix, with each sample’s genetic variants and their status (case or control) included. Statistical methods, such as logistic regression, are applied to each genetic variant to test for associations with the trait or disease of interest. Significant associations are identified based on statistical thresholds, highlighting potential genetic markers. To visualize the results, a Manhattan plot was generated using R, with significant SNP IDs labeled in the plot. This process helps in uncovering the genetic basis of complex traits and diseases.

The study used biochemical markers to compare stroke patients (mean age = 58) with healthy controls (mean age = 35). According to Table 7, there are notable variations between the two groups in both the serum lipid profile and blood glucose level. As a result, stroke patients had higher fasting blood sugar levels, glycated hemoglobin, cholesterol, LDL, VLDL, and triglycerides than healthy controls. Reduced HDL levels were also present in those whose lipid profiles had altered. The platelet count and liver enzymes ALT/AST did not differ substantially. Additionally, the statistical relationship between the gender and age of stroke patients and the importance of various allelic gene variations was examined.

**Table 7:** Comparative clinical characteristics of the study population.

Characteristic	Controls^a^	Patients^a^	*p* ^b^
Age	35.63 ± 12.81	58.44 ± 9.43	<0.001
Platelet count	235.9 ± 72.6	250.5 ± 84.3	0.171
Blood sugar fasting	98.97 ± 5.42	101.88 ± 5.41	<0.001
HbA1c	5.097 ± 0.402	6.027 ± 0.314	<0.001
Triglyceride	127.08 ± 9.42	165.9 ± 32.2	<0.001
Cholesterol	114.37 ± 8.20	171.2 ± 50.1	<0.001
HDL	48.1 ± 11.8	25.44 ± 3.57	<0.001
LDL	115.19 ± 9.65	148.4 ± 31.8	<0.001
VLDL	. 27.81 ± 5.40	43.3 ± 13.8	<0.001
AST	25.1 ± 10.3	27.7 ± 16.1	0.158
ALT	25 ± 10.2	30.7 ± 24.6	0.580

a Student’s *t*-test for continuous variables (variables with normal distribution).

b Values are presented as mean ± standard deviation.

### 3.1 Association of various genotypes with gender and age in stroke patients

**Figure 5:**
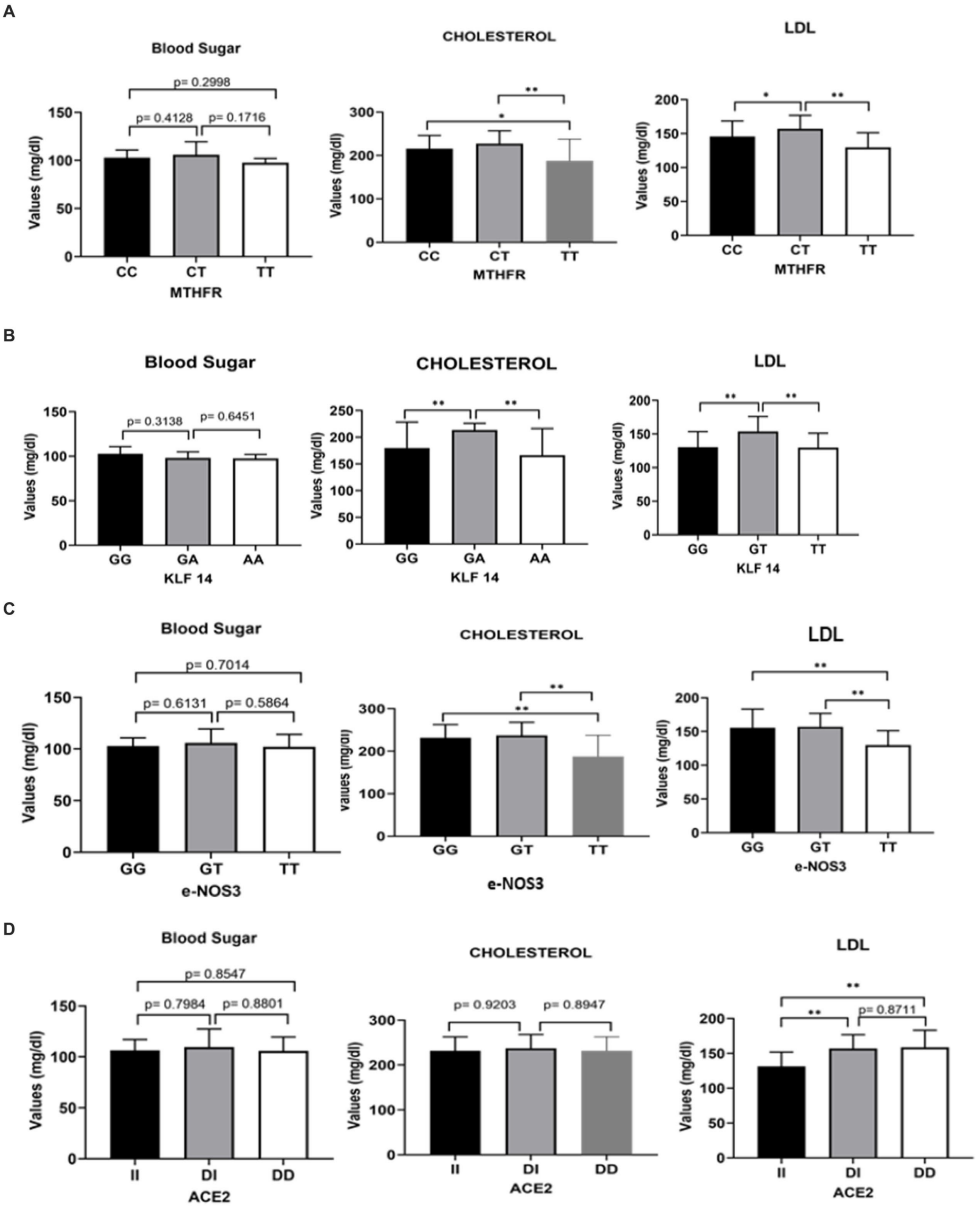
Clinical association of blood sugar, cholesterol and LDL with MTHFR, KLF 14, e-NOS3 and ACE2 genotypes in stroke patients. **(A)** Comparing *p-*value of blood sugar, cholesterol and LDL with MTHFR genotypes (CC/CT/TT). **(B)** Comparing the *p-*value of blood sugar, cholesterol and LDL with KLF14 genotypes (GG/GA/AA). **(C)** Comparing the *p*-value of blood sugar, cholesterol and LDL with e NOS3 genotypes (GG/GT/TT). **(D)** Comparing the *p-*value of blood sugar, cholesterol and LDL with ACE2 genotypes (II/DI/DD).

According to Table 5, the incidence of strokes in patients older than 50 years old and the number of patients of the male gender were higher than those of the female gender (Table 2) Our findings show that the KLF14 heterozygous genotype GA is more common in patients who are male (45.2%) than female (23.6%) and who are older (40%) than younger (25%) patients when compared to other genotypes. The heterozygous CT genotype was also more prevalent in patients over 50 (58.7%) compared to patients under 50 (26%) and in males (56.4%) compared to females (43.9%) in the case of the MTHFR polymorphism. In comparison to other genotypes, the genotype distribution for eNOS exhibited a larger distribution of GT heterozygosity in males (58%) and females (51.2%), as well as in patients who were >50 years of age (58.7%) and 50 years of age (43.5%) (Table 5). Similarly, the genotype distribution for ACE demonstrated a larger prevalence of DD homozygosity compared to other genotypes in males (69.3%) and females (58.5%), as well as in patients who were >50 years of age (68.7%) and 50 years of age (52.1%). The findings indicated that these genotypes may be linked to stroke risk and susceptibility independently or combined with other variables such as gender and age (Figure 5).

### 3.2 Comparative distribution frequency between stroke patients and controls (*p*-values) for KLF14 rs972283 G > a, MTHFR 677 C > T, eNOS3- rs1799983 G > T genotypes

KLF14 rs972283 G > A genotype prevalence in stroke patients was 28, 37, and 35%, compared to 39.16, 45, and 15% in healthy controls (Table 6). There was a statistically significant difference between stroke patients and controls in the KLF14 rs972283 G > A gene variation (*p*= 0.0004). Additionally, it was shown that stroke patients had a greater frequency of the allele A (0.53 vs. 0.38) than healthy individuals.

According to data in Table 6, the prevalence of MTHFR 677 C > T genotypes was reported as CC (40.77%), CT (51.45%), and TT (7.76%) in stroke patients, and CC (65.83%), CT (30.83%), and TT (3.33%) in controls. There was a statistically significant difference between stroke patients and controls in the MTHFR 677 C > T gene variation (*p* = 0.0008). Additionally, it was shown that stroke patients had a greater frequency of the allele T (0.33 vs. 0.19) than healthy individuals.

Table 6 shows our findings, which indicate that the frequency of the eNOS3- rs1799983 G > T genotypes in stroke patients was GG (36.89%), GT (55.33%), and TT (7.76%) and that it was GG (54.16%), GA (41.66%), and TT (4.16%) in healthy controls. The observed statistical significance (*p* = 0.030) of the eNOS3- rs1799983 G > T gene variant between stroke patients and controls was noted. Additionally, it was shown that stroke patients had a greater frequency of the allele T (0.35 vs. 0.25) than healthy persons.

According to Table 4, the prevalence of the ACE genotypes was II (16.66%), ID (45.83%), and DD (37.5%) in controls and II (12.62%), ID (22.33%), and DD (65%) in stroke patients (Table 6). Additionally, it was discovered that there was a statistically significant (*p* = 0.0001) distinction between stroke patients and controls in the ACE- rs4646994 I/D gene variation. Additionally, stroke patients exhibited a greater frequency of allele D (0.76 vs. 0.40) than healthy individuals.

### 3.3 Multivariate analysis to evaluate the association of polymorphic variants with the stroke risk

#### 3.3.1 KLF14 rs972283 G > A

The link between the KLF14 rs972283 G > A genotypes and the risk of stroke patients were examined using a multivariate analysis using logistic regression, such as an odds ratio (OD) or risk ratio (RR) with 95% confidence intervals (CI). Table 8 lists the results. With an OR of 3.09, (95%) CI = (1.492 to 6.407), RR = 1.78 (1.1915 to 2.662), and *p* = 0.0024, the codominant model showed a significant connection between the KLF 14-AA genotype and greater stroke susceptibility. There was a significant association between the KLF14 (GG + GA) and KLF14-AA genotypes, indicating increased stroke vulnerability, with OR = 2.86, RR = 1.72, and *p* = 0.003 in the recessive inheritance model. Additionally, it was shown that allele A had a strong correlation with the risk of having a stroke, with ORs of 1.85, RRs of 1.32, and *p* = 0.001.

**Table 8:** Multivariate analysis to evaluate the association of KLF14 G > A/ *MTHFR* 677 C > T /eNOS3 G > T/ ACE I/D genotypes with the ischemic Stroke risk.

Genotypes	Controls	Cases	OR (95% CI)	Risk ratio (RR)	*p*-value
Association of KLF14 G > A gene variation with stroke susceptibility					
**Codominant inheritance model**					
KLF14-GG	47	28	1 (reference)	1 (reference)	
KLF14-GA	54	37	1.15 (0.6140 to 2.154)	1.05 (0.8276 to 1.3476)	0.66
KLF14-AA	19	35	3.09 (1.4922 to 6.407)	1.78 (1.1915 to 2.662)	0.0024
**Dominant inheritance model**					
KLF14-GG	47	28	1 (reference)	1 (reference)	
KLF14-(GA + AA)	73	72	1.65 (0.9363 to 2.927)	1.24 (0.9811 to 1.579)	0.083
**Recessive inheritance model**					
KLF14 (GG + GA)	101	65	1 (reference)	1 (reference)	
KLF14-AA	19	35	2.86 (1.5099 to 5.4262)	1.72 (1.1802 to 2.5338)	0.003
**Allele**					
KLF14-G	148	93	1 (reference)	1 (reference)	
KLF14-A	92	107	1.85 (1.2649 to 2.708)	1.32 (1.1093 to 1.590)	0.001

Genotypes	Controls	Cases	OR (95% CI)	Risk ratio (RR)	*p*-value
Association of *MTHFR* 677 C > T gene variation with stroke susceptibility					
**Codominant inheritance model**					
MTHFR-CC	79	42	1 (reference)	1 (reference)	
MTHFR-CT	37	53	2.69 (1.5352 to 4.7287)	1.58 (1.2011 to 2.0999)	0.0006
MTHFR-TT	04	08	3.76 (1.0700 to 13.226)	1.95 (0.8708 to 4.4058)	0.038
**Dominant inheritance model**					
MTHFR-CC	79	42	1 (reference)	1 (reference)	
MTHFR-(CT + TT)	41	61	2.79 (1.6231 to 4.825)	1.62 (1.2399 to 2.1278)	0.0002
**Recessive inheritance model**					
MTHFR-(CT + TT)	116	95	1 (reference)	1 (reference)	
MTHFR-TT	04	08	2.44 (0.7134 to 8.3593)	1.64 (0.7341 to 3.7053)	0.15
**Allele**					
MTHFR-C	195	137	1 (reference)	1 (reference)	
MTHFR-T	45	69	2.18 (1.4134 to 3.370)	1.48 (1.1652 to 1.9002)	0.0004

Genotypes	Controls	Cases	OR (95% CI)	Risk ratio (RR)	*p*-value
Association of eNOS3- rs1799983 G > T gene variation with the risk of stroke					
**Codominant inheritance model**					
eNOS3-GG	65	38	1 (reference)	1 (reference)	
eNOS3-GT	50	57	1.95 (1.1231 to 3.3858)	1.35 (1.0513 to 1.7349)	0.018
eNOS3-TT	05	08	2.73 (0.8352 to 8.9683)	1.64 (0.8121 to 3.3150)	0.167
**Dominant inheritance model**					
eNOS3-GG	65	38	1 (reference)	1 (reference)	
eNOS3-(GT + TT)	55	65	2.02 (1.1807 to 3.4611)	1.37 (1.0785 to 1.7577)	0.010
**Recessive inheritance model**					
eNOS3-(GG + GT)	115	95	1 (reference)	1 (reference)	
eNOS3-TT	05	08	1.93 (0.6133 to 6.1166)	1.42 (0.7081 to 2.8629)	0.25
**Allele**					
G allele	180	135	1 (reference)	1 (reference)	
T allele	60	73	1.62 (1.0788 to 2.4395)	1.26 (1.0263 to 1.5634)	0.020

Genotypes	Controls	Cases	OR (95% CI)	Risk ratio (RR)	*p*-value
Association of ACE I/D polymorphism in the stroke patients and healthy controls					
**Codominant inheritance model**					
ACE-II	20	13	1 (reference)	1 (reference)	
ACE-ID	55	23	0.64 (0.2747 to 1.507)	0.85 (0.6302 to 1.172)	0.309
ACE-DD	45	67	2.29 (1.0355 to 5.066)	1.50 (1.0566 to 2.153)	0.040
**Dominant inheritance model**					
ACE-II	20	13	1 (reference)	1 (reference)	
ACE-(ID+DD)	100	90	1.38 (0.6514 to 2.943)	1.15 (0.8476 to 1.564)	0.39
**Recessive inheritance model**					
ACE-(II + ID)	75	36	1 (reference)	1 (reference)	
ACE-DD	45	67	3.10 (1.7926 to 5.367)	1.50 (1.0566 to 2.1534)	0.0001
**Allele**					
ACE-I	95	49	1 (reference)	1 (reference)	
ACE-D	145	157	2.09 (1.3903 to 3.1697)	1.37 (1.1640 to 1.6220)	0.0004

#### 3.3.2 MTHFR 677 C > T

These findings showed that the MTHFR-677 CT genotype was significantly related to greater stroke vulnerability in the codominant model, with an OR of 2.69, RR = 1.58, and *p* = 0.0006 (Table 7). The dominant inheritance model shows a robust connection between the MTHFR-CC and MTHFR-(CT + TT) genotypes, which increases stroke risk with an OR = 2.79, RR = 1.62, and *p* = 0.0002. However, in the recessive model, OR = 2.44, RR = 1.64, and *p* = 0.15 did not indicate a connection between the MTHFR-(CT + TT) and MTHFR-TT genotypes. Further analysis reveals that the allele T, with an OR of 2.18, RR of 1.48, and *p* = 0.0004, was significantly linked with stroke susceptibility.

#### 3.3.3 eNOS3- rs1799983 G > T

The analysis’s findings showed a high connection between the eNOS3-GG and eNOS3-GT genotypes with an elevated risk of ischemic stroke, with OR = 1.95, RR = 1.35, and *p* = 0.018, in the codominant model (Table 7) With an OR = 2.73, RR = 1.64, and *p* = 0.167, we were unable to detect any correlation between the eNOS3-GG and eNOS3-TT genotypes and the risk of stroke. The dominant inheritance model used in this investigation revealed a substantial connection between the eNOS3-GG and eNOS3-(GT + TT) genotypes and an elevated risk of stroke, with OR = 2.02, RR = 1.37, and *p* = 0.010. However, in the recessive inheritance model with stroke susceptibility, no correlation was found between the eNOS3 (GG + GT) and eNOS3-TT genotypes with OR = 1.93, RR = 1.42, and *p* = 0.25. In addition, an allelic analysis revealed that the allele T had a high OR of 1.62, RR of 1.26, and *p* = 0.020 correlated with stroke susceptibility.

#### 3.3.4 ACE- rs4646994 I/D

Our findings showed that the ACE-DD genotype was significantly related to greater stroke susceptibility in the codominant model, with an OR of 2.29, RR = 1.50, and *p* = 0.040. A significant connection between the ACE-DD and ACE-(II + DD) genotypes and an elevated risk of stroke was also found in the recessive inheritance model, with OR = 3.10 (95%) CI (1.7926 to 5.367), RR = 1.50 (1.0566 to 2.1534), and *p* = 0.0001 (Table 8). With an OR of 2.09 (95% CI) (1.3903 to 3.1697), RR of 1.37 (1.1640 to 1.6220), and *p* = 0.0004, the D allele was significantly related to stroke susceptibility in allelic comparison.

### 3.4 Association of blood sugar, cholesterol and LDL with MTHFR 677 C > T, eNOS3- rs1799983 G > T genotypes, KLF14 rs972283 G > a, ACE- rs4646994 I/D genotypes in ischemic stroke patients

In Figure 3, MTHFR, KLF 14, e-NOS3, and ACE2 were used to compare the significant laboratory traits of blood sugar, cholesterol, and low-density lipoprotein (LDL) in individuals with ischemic stroke illness. Stroke patients with the CT genotype of MTHFR and the GA genotype of KLF14 were shown to have significantly higher cholesterol and LDL expression results. Comparatively to TT alleles, patients with the e-NOS3 GG and GT genotypes had highly significant outcomes. According to the LDL results, as shown in Figure 3, both the DI and DD genotypes had higher levels of ACE2 than the II genotype.

## 4 Discussion

Despite the rapid development of new genetic research methods, we still need to learn more about detecting Mendelian ischemic stroke variations. Whole-exome sequencing screening utilizing a comprehensive stroke gene panel may uncover rare monogenic causes of ischemic stroke. At the same time, careful assessment of clinical characteristics and possible toxicity of new alterations must still be done.

### 4.1 Whole exome sequencing (WES), LDLR, and stroke

We performed WES on ischemic stroke patients to uncover unusual monogenic types of ischemic stroke. Using a comprehensive panel of stroke genes, we looked for alterations in the genes associated with ischemic stroke. The clinical stroke subtype of the probands was related to characteristics previously connected to harmful mutations in these genes (Figures 1, 2 and Tables 3, 5).

#### 4.1.1 Low-density lipoprotein receptor (LDLR)

On chromosome 19 (19p13), the 45 kb long LDLR gene, which has 18 exons, encodes LDLR proteins. The binding and endocytosis of plasma LDL particles from the blood circulation are carried out by the LDLR, a cell surface glycoprotein. It primarily aids in keeping cellular cholesterol homeostasis in check (Go and Mani, 2012). Polymorphic mutations in the LDLR gene, linked to a higher risk of atherosclerosis, can potentially increase plasma LDL levels (Muallem et al., 2007) considerably. There have been reports of several LDLR gene mutations affecting the promoter regions, splicing sites, and exons. Because of these gene variations, familial hypercholesterolemia can develop (Strom et al., 2014). Through whole exome sequencing, three genes—LDLR, APOB, and PCSK9—have recently been connected to stroke vulnerability (Tung et al., 2021). According to Tung et al. (2021), patients with ischemic stroke who carry FH pathogenic mutations were more likely to have a significant artery stroke and a transient ischemic attack. LDLR, LDLRAD2, and LDLRAD3 gene variations were discovered using whole exome sequencing in our stroke patients. In all of our stroke patients, whole exome sequencing has found the following gene variations to be the most prevalent: LDLR rs5927 (c.2232A > G), LDLRAD3 rs1138807 (c.219A > G), and LDLRAD2 rs10917051 (c.401A > C) Table 2. Independent of gender, low-density lipoprotein cholesterol and CAD are linked by LDLR exon 12 rs688 (Martinelli et al., 2010). The LDLR rs688 gene variant has been described by Jha et al. (2018) as a hereditary risk factor for CAD in Indian population. Two more LDLR gene variations (rs5925 and rs1529729), according to another Indian study, have been linked to an increased risk of CAD (Jha et al., 2019). The LDLR gene is mutated in more than 93% of FH patients (Usifo et al., 2012). A more severe phenotype of homozygous (HoFH) and autosomal recessive FH that differs by impacted gene and residual enzyme function can be seen in people with biallelic pathogenic mutations (either homozygous or compound heterozygous variations) in APOB, LDLR, PCSK9, or LDLRAP1 (McGowan et al., 2019). Any ischemic, large artery, and small vessel stroke were related to higher risk when apoA-I and HDL cholesterol levels were low (Hindy et al., 2018). Recent research shows a causal relationship between elevated LDL cholesterol, triglycerides, and apoB and an increased risk of ischemic stroke (Sun et al., 2019), and a more extraordinary relationship between apoB and cardiovascular disease (Brunner et al., 2019). According to whole exome sequencing, our stroke patients had numerous gene variations in the APOA2, APOA3, and APOA4 genes. WES was used to identify the insertion/deletion gene variants APOA2- rs17244502 (c.152-11_152-6del), APOA3- rs5092 (c.87G > A), and APOA4- rs5104 (c.440G > A). Few studies compare how LDL cholesterol, triglycerides, and apoB affect stroke risk.

### 4.2 Whole exome sequencing (WES) and ischemic stroke

Zhang et al.’s study (Zhang et al., 2014) found a connection between the MTHFR gene A1298C polymorphism and the onset of ischemic stroke specially in Asian population. Additionally, they mention MTHFR A1298C, which may predict clinical progression and increase the risk of ischemic stroke (Zhang et al., 2014). According to a Chinese population-based study (Jiang et al., 2014), MTHFR polymorphisms may be essential in the genetic determination of serum lipid levels in chinse hypertensive patients (Jiang et al., 2014). In our stroke patients, frequent gene variants in the MTHFR gene were discovered using WES screening and a comprehensive stroke gene panel (Figures 1, 2 and Tables **3**, 5). The most prevalent MTHFR gene variations discovered through whole exome sequencing in all of our ischemic stroke patients are MTHFR rs750510348 C > T (c.1428C > T), MTHFR- rs2274976 (c.1904G > A), MTHFR rs2066462 (c.1179C > T), and MTHFR rs2066470 (c.240C > T) (Table 5). Similar to this, the three most prevalent eNOS3 gene variations found by WES in all of our stroke patients are NOS3rs1549758 (c.774 T > C), NOS3rs1799983 (c.894 T > G), and NOS3rs2566514 (c.1998C > G) (Table 5). KLF14 rs111400400 (c.172C > T), KLF14 rs184537657 (c.140C > A), and KLF14 rs111731678 (c.117 T > A) are the most prevalent KLF14 gene variants found by whole exome sequencing in all of our stroke cases (Table 5). ACE- rs4362 (c.3387 T > C), ACE- rs4343 (c.2328G > A), and ACE- rs4309 (c.1215C > T) are the most prevalent ACE gene variations found by whole exome sequencing in all of our ischemic stroke patients (Table 5). These genetic variants, which are also linked to the ethnic makeup of a particular human group, may operate as a standalone risk factor for stroke or have an impact on other modifiable and non-modifiable risk factors as well as the pathophysiological mechanisms that underlie it (Yang and Civelek, 2020). A tailored approach to preventive and therapeutic measures has been supported by evidence from studies concentrating on allele-specific effects that increase a person’s chance of contracting a disease (Li et al., 2018). These result comes in line with studies reported that association of the eNOS3, KLF14 and ACE with metabolic syndrome (Mir et al., 2022a; Akash et al., 2023; Elfaki et al., 2023; Yang and Civelek, 2020; Xi et al., 2012; Elfaki et al., 2021). The Metabolic syndrome is composed of obesity, hyperglycemia, dyslipidemia, and hypertension, insulin insensitivity and is risk factors for atherosclerosis, and Type 2 diabetes (T2D) and cardiovascular disease (Won et al., 2013; Aboonabi et al., 2019; Bayturan et al., 2010).

#### 4.2.1 GSTT4 novel gene variants

Results identified 11 novel gene variants in GSTT4 gene SNPs (Table 6), GSTT4c.699 T > C, GSTT4-c.701C > G, GSTT4-c.708G > T, GSTT4-c.710 T > G, GSTT4-c.712A > G, GSTT4-c.712A > G, GSTT4-c.718A > T, GSTT4-c.719G > A, GSTT4-c.721A > T, GSTT4-c.722G > T, one deletion GSTT4p.Asn232LysfsTer6, one insertion GSTT4c.688_689insCG and in the ischemic stroke patients. These results of GSTT4 require performing case-control studies to examine the effect of these gene variants in identifying individuals susceptible to stroke. This result is consistent with.

#### 4.2.2 Association of Krüppel-like factor-14 rs972283 G > a with ischemic stroke

The rs972283 polymorphism in KLF14 has previously been strongly linked to an increased risk of T2D in the global population, with a population attributable risk percentage of 4.18% (Wang et al., 2014). This association has been supported by a meta-analysis of five studies including 50,552 cases and 106,535 controls. Recently, it was reported that the KLF14 rs4731702 variant and the related linkage disequilibrium polymorphisms may have a role in sex-, age-, and obesity-dependent cardiometabolic disorders (Wu et al., 2022). Additionally, it has been demonstrated that the KLF14 rs4731702 SNP substantially correlates with serum lipids as a predictor of cardiovascular disease (Huang et al., 2013). These studies report significant correlation between the incidence of stroke and the KLF14 rs972283 G > A polymorphic gene variant as found by our study (Tables 7, 8 and Figure 3B). Our results showed that the AA genotype of the KLF14 rs972283 G > A SNP was associated with stroke. Results also showed that there is a significant difference in plasma cholesterol between KLF14 rs972283 G > A SNP genotype (Figure 3B). The KLF14 is regarded as a master regulator of gene expressions in adipose tissues and that many of its polymorphisms are connected with obesity, T2D, and altered lipid profiles in people, all of which are risk factors for stroke (Elfaki et al., 2023; Wang et al., 2014; Yin et al., 2013).

#### 4.2.3 Association of the MTHFR C677T and ischemic stroke

The functional polymorphism MTHFR C677T, which changes the amino acid alanine to the amino acid valine at codon 222 of the encoded protein, has the potential to drastically effect on the activity of the MTHFR enzyme (Frosst et al., 1995). According to previous studies, people with the Val/Val genotype (TT) have enzyme activity that is decreased to 30% of regular activity, and heterozygotes (CT) have activity reductions that are also significant at 65% of regular enzyme activity (Nazki et al., 2014; Zhao et al., 2017). The reduced MTHFR enzyme activity, which raises total plasma homocysteine levels and lowers plasma folate levels, has been suggested to have a role in the pathogenesis of stroke (Panigrahi et al., 2006). According to the available data, MTHFR homozygosity C677T is strongly associated with an increased risk of thrombotic events and ischemic stroke (Dionisio et al., 2010; Kelemen et al., 2004). The frequency of ischemic stroke has also been associated with polymorphic gene polymorphisms such as MTHFR 2572C > A and 6,685 T > C (Hartl et al., 2023). These studies are in agreement with our result that showed the TT genotype of the SNP MTHFR C677T are associated with plasma cholesterol (Tables 7, 8 and Figure 3A).

#### 4.2.4 Association of the endothelial nitric oxide synthase (eNOS)

rs1799983 and ischemic stroke—Nitric oxide (NO), a substance generated by vascular endothelial cells that regulate regional blood flow and blood pressure. It has been speculated that the endothelial nitric oxide synthase (eNOS) G894T gene polymorphism increases the risk of developing essential hypertension (EH), although the findings are still debatable. Additionally, according to our analysis, the allele T was substantially related to stroke susceptibility in our population, with an OR of 2.18. Endothelial nitric oxide synthase (eNOS) genes are essential for controlling blood pressure. Their activity has been linked to hypertension, a known risk factor for the stork, as altered nitric oxide levels in endothelial cells significantly alter blood pressure through their effects on vasodilation, platelet aggregation, leukocyte adhesion, respectively, and smooth muscle cell proliferation (Wang et al., 2014). According to studies, nitric oxide levels were reduced, and blood pressure elevated when the eNOS gene was inhibited in healthy people (Gamboa et al., 2007). A strong connection between the eNOS rs1799983 polymorphism and the risk of hypertension under multiple genetic models was recently established by a meta-analysis of studies encompassing 14,185 cases and 13,407 controls (Panigrahi et al., 2006). Another meta-analysis that included 27 case-control studies with 6,733 cases and 7,305 controls also discovered a significant link between the eNOS G894T SNP and ischemic stroke (Shi et al., 2021). In the dominant inheritance paradigm, we have found a substantial correlation between the eNOS3-GG and eNOS3-(GT + TT) genotypes and an elevated risk of stroke. These studies are consistent with the current study demonstrated that the GT genotype of the eNOS3- rs1799983 are associated with blood cholesterol (Tables 7, 8 and Figure 3C).

#### 4.2.5 Association of the ACE insertion/deletion I/D and ischemic stroke

A key component of the renin-angiotensin system, angiotensin-converting enzyme (ACE) is engaged in vascular remodeling by accelerating the conversion of angiotensin I to angiotensin II, which has a vasoconstrictive impact (Gamboa et al., 2007). There has been evidence of an I/D polymorphism (rs4646994- I/D) in the ACE gene’s intron 16 based on the presence or absence of a DNA fragment (287 bp). It has been suggested that these variations may affect a population’s susceptibility to certain diseases, such as CAD, T2D, and ischemic stroke (Elfaki et al., 2021; Hemeed et al., 2020; Kumar et al., 2017). It has been reported that patients with ischemic stroke have significantly greater rates of the ACE-DD genotype (37.8%) and D allele (57.3%) than people without the condition (Kim and Iwao, 2000). Interestingly, a meta-analysis of risk estimations with 10,070 stroke cases and 22,103 healthy controls showed a 37% higher risk of ischemic stroke linked with the DD homozygote than II homozygotes and ID heterozygote with an odd ratio of 1.37 (Hemeed et al., 2020). Strong evidence linking the DD genotype to an elevated risk of ischemic stroke has been found in studies using dominant and recessive models (Zhang et al., 2012; Salem and Gab-Allah, 2020). As demonstrated in this investigation, the ACE-DD genotype was significantly related to enhanced stroke susceptibility in both codominant and recessive inheritance models, with odd ratios of 2.29 and 3.10, respectively (Tables 7, 8). The ACE I/D polymorphism was also associated with blood lipids (Figure 3D). It may play a role in risk and pathogenesis in the population under consideration. Future research are important to examine genetic biomarkers in ischemic stroke susceptibility, etiology, and prognosis in the individualized approach to prevention, treatment, and therapy (Yamamoto et al., 2022).

## 5 Limitations

This study has several limitations that should be considered when interpreting the findings. First, the sample size was relatively modest, which may have limited the statistical power to detect rare genetic variants or subtle associations. Second, functional assays were not performed to confirm the biological effects of the identified GSTT4 and other gene variants. Future studies will include *in vitro* and *in vivo* functional validation, such as recombinant protein expression and enzymatic activity assays, to elucidate the mechanistic role of these GSTT4 variants in oxidative stress and stroke susceptibility. Third, the study cohort was limited to Saudi nationals from the Tabuk region; therefore, the findings may not be generalizable to other populations. Although all participants were Saudi nationals from the same geographic area, potential population stratification cannot be entirely excluded; these findings are specific to the Saudi population from the Tabuk region and may not be directly generalizable to other ethnic groups or geographic areas due to possible genetic and environmental differences. Future studies will incorporate ancestry-informative markers (AIMs) and principal component analysis (PCA) to control for subtle genetic substructure and reduce the risk of false-positive associations. Finally, detailed clinical and environmental data, such as stroke subtypes, comorbidity severity, lifestyle factors, and treatment history, were not available for all participants.

To address these limitations, future multicenter studies with larger, ethnically diverse populations and experimental validation are recommended to confirm and expand these observations and to validate the identified variants across broader cohorts. Future research will also integrate more comprehensive clinical datasets to strengthen genotype–phenotype correlation analyses and enhance the clinical applicability of these findings.

## 6 Conclusion

This study identified novel GSTT4 variants and other genetic alterations potentially associated with ischemic stroke in the Saudi Tabuk population. These findings highlight the contributions of oxidative stress, endothelial dysfunction, and lipid metabolism pathways to stroke susceptibility, underscoring the need for functional validation and larger multicenter studies. Taken together, this case-control study identified 11 novel variants in the GSTT4 gene, one deletion, one insertion, and nine SNVs, along with intermediate and common variants in cholesterol-associated genes (LDLR, LDLRAD2, LDLRAD3, APOA2, APOA3, APOA4, APOA5, PCSK9) and vascular-regulatory genes (MTHFR, KLF14, eNOS3, and ACE). The alleles A of KLF14, T of MTHFR, and T of eNOS3, as well as D of ACE, may contribute to stroke susceptibility in the Tabuk population. These findings highlight population-specific genetic factors that warrant validation through large-scale case-control studies and functional protein analyses to enhance risk stratification for ischemic stroke.

## Data Availability

The original contributions presented in the study are included in the article/supplementary material, further inquiries can be directed to the corresponding author/s.
